# De Novo L509P Mutation of the *TGFBI* Gene Associated with Slit-Lamp Findings of Lattice Corneal Dystrophy Type IIIA

**DOI:** 10.3390/jcm11113055

**Published:** 2022-05-28

**Authors:** Yong Woo Ji, Hyunmin Ahn, Kyoung-Jin Shin, Tae-im Kim, Kyoung Yul Seo, R. Doyle Stulting, Eung Kweon Kim

**Affiliations:** 1 Department of Ophthalmology, Yongin Severance Hospital, Yonsei University College of Medicine, Yongin 16995, Korea; lusita30@yuhs.ac; 2 Department of Ophthalmology, Institute of Vision Research, Severance Hospital, Yonsei University College of Medicine, Seoul 03722, Korea; overhyun31@gmail.com (H.A.); tikim@yuhs.ac (T.-i.K.); seoky@yuhs.ac (K.Y.S.); 3 Department of Forensic Medicine, Yonsei University College of Medicine, Seoul 03722, Korea; kjshin@yuhs.ac; 4 Corneal Dystrophy Research Institute, Yonsei University College of Medicine, Seoul 03722, Korea; 5 Woolfson Eye Institute, Atlanta, GA 30328, USA; dstulting@woolfsoneye.com; 6 Saevit Eye Hospital, Goyang 10447, Korea

**Keywords:** de novo mutation, tautomeric shift, Leu509Pro (L509P), lattice corneal dystrophy, transforming growth factor-β-induced (*TGFBI*) gene

## Abstract

Background: Mutations of the transforming growth factor-β-induced (*TGFBI*) gene produce various types of corneal dystrophy. Here, we report a novel de novo L509P mutation not located in a known hot spot of the transforming growth factor-β-induced (*TGFBI*) gene and its clinical phenotype, which resembles that of lattice corneal dystrophy type IIIA (LCD IIIA). Case presentation: A 36-year-old man (proband) visited our clinic due to decreased visual acuity with intermittent ocular irritation in conjunction with painful recurrent erosions in both eyes for 10 years. Molecular genetic analyses revealed a *TGFBI* L509P mutation (c.1526T>C) in the proband and one of his sons. Interestingly, neither *TGFBI* mutations nor corneal abnormalities were detected in either of the proband’s biological parents, indicating the occurrence of a de novo L509P mutation. Clinical examinations, including slit-lamp retro-illumination and Fourier-domain anterior segment optical coherence tomography (FD-OCT), revealed gray deposits in the anterior stroma and deeper refractile lines extending from limbus to limbus in both corneas of the proband, consistent with a diagnosis of LCD IIIA. Superficial diffuse haze and surface irregularity were observed in conjunction with corneal erosions and visual impairment, necessitating phototherapeutic keratectomy (PTK). A 60 μm PTK of the Bowman layer and anterior stroma of the proband’s left eye was performed following the removal of the epithelium in order to remove superficial corneal opacities. His BCVA improved from 20/400 to 20/50 at postoperative week 8 and was maintained for 45 months. Pinhole-corrected VA was 20/20 at the last visit, and corneal opacities had not recurred. Conclusions: An inheritable de novo mutation of L509P in the *TGFBI* gene can produce severe LCD IIIA, which can be successfully treated with OCT-guided PRK.

## 1. Background

Transforming growth factor beta-induced protein (TGFBIp) is an extracellular matrix protein that is highly conserved among vertebrate species such as humans, chimpanzees, mice, zebrafish, and western clawed frogs [1,2]. Discovered during a search for genes induced by TGF-β, it is encoded by the *TGFBI* gene, which has been mapped to chromosome 5q31. TGFBIp is found in several tissues of the human body, though the expression of this protein is clinically significant only in the cornea. Mutations of the *TGFBI* gene in humans induce the abnormal accumulation of insoluble TGFBIp in the cornea. Though such mutations are associated with differing phenotypes, the resultant conditions are collectively referred to as *TGFBI*-related corneal dystrophies [3,4,5].

One such condition, known as lattice corneal dystrophy (LCD), is characterized by a network of multiple branching refractile lines in the anterior to mid-stromal level in its amyloidogenic form. Most LCDs are related to *TGFBI* mutations that arise from the fourth fasciclin domain (FAS 1–4) of *TGFBI* [4,6]. L509P mutations of the *TGFBI* gene are rare and are associated with a variety of phenotypes. In one German family, the clinical phenotype of the L509P mutation in question resembled Reis–Bücklers corneal dystrophy (RBCD) without lattice lines [7]. In contrast, the phenotype and histology resembled that of LCD in a French family [8]. More recently, Lisch et al. recently reported a case of LCD type 1 associated with an L509P mutation [9].

Spontaneous de novo mutations, which are not detected in the parents, are rare, with an incidence of 10^−4^ to 10^−6^ per gene per generation in eukaryotes and 10^−5^ to 10^−7^ per gene per generation in bacteria and phages [10,11,12,13]. Once these mutations occur, however, they can then be passed to future generations, as spontaneous mutations typically occur in the germline [13,14]. Several mutations of the *TGFBI* gene have been previously reported: R124L, G→T; R555Q, G→A; R124C, C→T; A546D, C→A [15,16,17]. R124 and R555 have been identified as mutation “hot spots” on the *TGFBI* gene [18]. De novo mutations more frequently involve a transition from G:C to A:T than from A:T to G:C [19,20].

Herein, we report for the first time the occurrence of a heritable, de novo mutation of L509P of the *TGFBI* gene in a patient exhibiting clinical signs of LCD IIIA. Therefore, clinicians must remain aware of the possibility that de novo mutations can give rise to *TGFBI*-related corneal dystrophy, even when an individual’s parents exhibit normal phenotypes.

## 2. Case Presentation

### 2.1. Clinical Analysis

**Figure 1:**
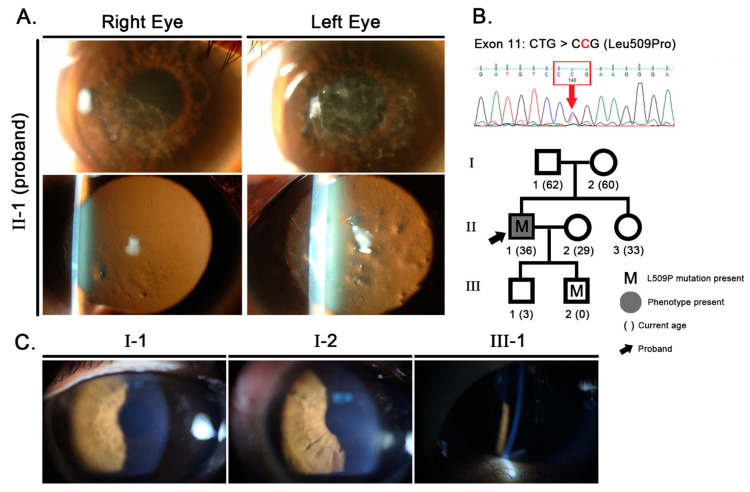
Slit-lamp photographs and molecular genetic analyses. (**A**) Slit-lamp examination revealed diffuse opacities in the superficial corneal stroma and deeper lattice lines in both corneas of the proband. (**B**) Partial nucleotide sequences of exon 11 of the transforming growth factor-β-induced (*TGFBI*) gene displayed a heterozygous T→C transition at nucleotide 1526 in the affected individual, leading to the change of the normal leucine residue to a proline residue (Leu509Pro, L509P). This mutation was not observed in any other family members, including the biological parents, but was detected in the younger son of the proband (III-2). (**C**) The proband’s biological parents and both of his sons had normal corneas. (Patient III-2 was too young for slit-lamp photographs to be obtained).

A 36-year-old man (proband, Patient II-1) visited our clinic due to decreased visual acuity in both eyes with a clinical diagnosis of RBCD made at another clinic. His best-corrected visual acuity (BCVA) was 20/50 OD and 20/400 OS at that time. The patient also had experienced intermittent ocular irritation in conjunction with painful recurrent erosions for 10 years but had no other significant ocular history and had not undergone any ocular surgery. His corneas exhibited an irregular surface and diffuse grayish-white deposits in the subepithelial stroma, along with distinct refractile lines spreading to the periphery with retro-illumination (Figure 1), resembling the LCD IIIA phenotype. His left eye exhibited more severe slit-lamp findings with poorer vision than the right eye. Slit-lamp examination of his parents, sister, and elder son (III-1) revealed clear, healthy corneas (Figure 1). No corneal opacities were observed in the younger son (III-2), who was 10 months old at the time of examination. (Detailed methodology of the research was described in Appendix A).

**Figure 2:**
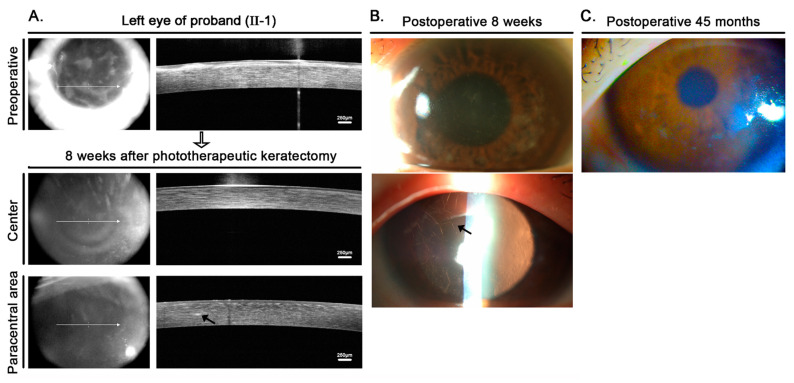
FD-OCT images and slit-lamp photographs of the left eye of the proband (II-1) after phototherapeutic keratectomy. (**A**) Preoperative Fourier-domain anterior segment optical coherence tomography (FD-OCT) images showing dense, diffuse, thick anterior stromal opacities of the left eye of the proband. At 8 weeks after phototherapeutic keratectomy (ablation depth: 60 μm), anterior stromal haze was no longer visible, but lattice lesions (black arrow) remained in the paracentral stroma about 230 μm from the posterior corneal surface. (**B**) Slit-lamp examination at 8 weeks showed central clearing, with persistent peripheral lattice lines consistent with the FD-OCT images. (**C**) Forty-five months postoperatively, slit-lamp examination showed no significant recurrence of the anterior stromal opacities.

Due to his level of visual impairment, a 60 μm phototherapeutic keratectomy (PTK) of the Bowman layer and anterior stroma of the proband’s left eye was performed following the removal of the epithelium in order to remove superficial corneal opacities. Fourier-domain anterior segment optical coherence tomography (FD-OCT) (RTVue-100; Optovue Inc., Fremont, CA, USA) confirmed the removal of superficial opaque deposits following PTK (Figure 2), but deeper linear opacities in the mid-periphery located 230 μm from the posterior corneal surface that could be seen by retroillumination remained (Figure 2). His BCVA improved from 20/400 to 20/50 at postoperative week 8 and was maintained for 45 months, with −2.75 diopters as the spherical equivalent for refractive error. Pinhole-corrected VA was 20/20 at the last visit, and corneal opacities had not recurred.

### 2.2. Molecular Genetic Analysis

Molecular analysis of all exons of the *TGFBI* gene revealed that the proband (II-1) had a single missense mutation, which is a heterozygous T→C transition at nucleotide 1526 of exon 11, leading to the change of a residue from leucine to proline (c.1526T>C, L509P, Leu509Pro) (Figure 1). One homozygous polymorphism (c.651G>C of exon 6, L217L) was detected concurrently, but this has also been found in normal individuals (data not shown). The lack of other *TGFBI* mutations associated with corneal opacities and the presence of a single FAS 1–4 point mutation, L509P, previously associated with LCD, supports our conclusion that the L509P mutation is the cause of corneal opacities in our reported case. Since all *TGFBI* mutations are known to exhibit autosomal dominant inheritance, exon 11 of the *TGFBI* genes of family members were examined. Neither of the proband’s parents had a mutation, but one of his sons had an L509P mutation of the *TGFBI* gene (Figure 1).

### 2.3. Confirmation of Paternity

Because we did not find mutations in the parents of the proband, we suspected that the heterozygous L509P observed in the proband might have been derived from a de novo mutation. We performed genetic analysis to look for shared autosomal short tandem repeat (A-STR) markers on DNA samples of the proband and his parents, which is a method commonly used to test for paternity in forensic science. The calculated probability of paternity for the family was higher than 99.99%, which is sufficient to support the conclusion that the parents of the proband are actually his biological father and mother. 

## 3. Discussion and Conclusions

Corneal dystrophies associated with inherited mutations of the *TGFBI* gene are common, but de novo mutations of the *TGFBI* gene are rare. To our knowledge, this is the first report of a de novo L509P mutation of the *TGFBI* gene and the fifth to discuss any form of de novo mutation of *TGFBI* [15,16,17]. Previously reported de novo mutations included the transversion of G→T at the R124L hot spot, the transition of G→A at R555Q, the transition of C→T at R124C, and the transversion of C→A transition at the non-hot spot of A546D, all of which have been documented as G:C→A:T transitions [15,16,17]. The L509P (c.1526T>C) mutation of the present case, however, occurred outside of known hot spots and exhibited an unusual T→C transition mutation, the exact mechanism of which remains undetermined.

DNA bases typically occur in many forms known as tautomers or structural isomers, which differ in the positions of their atoms and in the bonds between atoms. Adenine and cytosine exist in both amino and imino forms, and research has indicated that C transforms into the rare imino form to be paired with A. In the c.1526T>C de novo mutation of the present case, A would have undergone a tautomeric shift to its imino form during replication, which would have then paired with C (proband with de novo mutation). The result is a T→C mutation relative to the original molecule.

Using molecular genetic analysis, we detected an L509P mutation in one of the proband’s offspring (III-2), who was 10 months old, but no opacities were observed in his corneas. At the present time, this case does not provide evidence for Mendelian-dominant transmission of the phenotype as seen with previously reported L509P mutations; however, it is possible (and perhaps probable) that the son of the proband will develop LCD later in life.

Even though cases of an L509P mutation are rare, three groups of researchers have reported phenotypes associated with these mutations. Gruenauer-Kloevekorn et al. reported that a family of German patients with an L509P mutation exhibited a clinical phenotype resembling RBCD, though histology was not consistent with this phenotype [7]. They further stated that the L509P phenotype was not identical to those diagnosed with LCD since no lattice lines were observed. In contrast, Niel-Butschi et al. reported that their French patients with L509P mutations exhibited phenotypes consistent with LCD, with fine lattice lines in the deeper stroma [8]. Niel-Butschi et al. also identified another family with an L509R mutation associated with prominent lattice lines, implying that mutations affecting the Leu509 residue are amyloidogenic, thereby giving rise to lattice lines [8]. Lisch and Seitz further reported the case of a German family with an L509P mutation exhibiting an LCD phenotype [9]. In the present case, we observed a de novo mutation associated with an LCD IIIA phenotype, including the presence of deep lattice lines.

Interestingly, both the first reported case of corneal opacities associated with the L509P mutation and our case were initially diagnosed as RBCD. We suspect that the lattice lines in our case may not have been recognized by his previous physician. Our patient was only 26 years of age, suggesting that lattice lines may not appear until after the subepithelial opacities in cases with the L509P mutation. This could not, however, explain the case reported by Gruenauer-Kloevekorn et al., who underwent keratoplasty during his fifth decade of life.

Our patient exhibited poor visual acuity and eventually required surgical treatment. It is well-known that PTK can be effective for patients with granular corneal dystrophies and LCDs who have superficially accentuated opacities prior to keratoplasty [21,22]. In the present study, FD-OCT was undertaken in order to estimate the ablation depth that would be required to remove superficial opacities prior to PTK. The removal of 60 μm of superficial opacities increased the proband’s visual acuity from 20/400 to 20/50. The deep lattice opacities, however, could not be removed by PTK. Chen et al. reported that significant recurrence was observed at an average of 19.7 months (6–46 months) following treatment of LCD with PTK [23]. The proband of the present case exhibited neither a decrease in visual acuity nor significant recurrence 45 months after PTK, suggesting that the long-term outcome of PTK for patients with the L509P mutation may be better than they are with classical LCD.

In conclusion, the present report is the first to document a de novo L509P *TGFBI* mutation. The patient exhibited the definite clinical characteristics of LCD IIIA as well as molecular genetic inheritance, even in the absence of known parental family history. When suspected cases of LCD are encountered, clinicians must consider the possibility of de novo mutations, utilizing genetic testing to confirm or identify mutations. PTK may be effective in increasing visual acuity and delaying keratoplasty.

## Data Availability

All data analyzed during the current study are available from the corresponding author (E.K.K.) on reasonable request.

## Abbreviations

Transforming growth factor beta-induced, *TGFBI*; lattice corneal dystrophy, LCD; Fasciclin domain, FAS; Reis–Bücklers corneal dystrophy, RBCD; best corrected visual acuity, BCVA; phototherapeutic keratectomy, PTK; Fourier-domain anterior segment optical coherence tomography, FD-OCT; autosomal short tandem repeat, A-STR.

## Appendix A. Materials and Methods

### Appendix A.1. Patients and Clinical Evaluation

The proband (II-1) and his family members visited our clinic in September 2014 and were examined until June 2019 at Yonsei University Medical Center, Seoul, Korea. The present study was performed in accordance with the Declaration of Helsinki and approved by the Severance Hospital Institutional Review Board (No. 2016-1067-001). Written informed consent was obtained from all family members prior to their participation in the study.

Slit-lamp photography (D2X; Nikon Corporation, Tokyo, Japan) and Fourier-domain anterior segment optical coherence tomography (FD-OCT) (RTVue-100; Optovue Inc., Fremont, CA, USA) were performed in order to identify disease characteristics and assess the depth of corneal lesions. Phototherapeutic keratectomy (PTK) was performed on the cornea of the proband’s left eye for diffuse haze removal using the VISX S4 IR (VISX Inc., Santa Clara, CA, USA), as previously described [22,24]. Briefly, after the initial 30-µm PTK, the density and depth of the remaining diffuse stromal haze were assessed using the slit-lamp during laser ablation every 10-µm to prevent over-ablation.

### Appendix A.2. Genomic DNA Preparation and Mutation Analysis

Transforming growth factor beta-induced (*TGFBI*) gene was prepared and analyzed in all family members, as previously described [25,26,27,28]. Briefly, 200 μL of blood was drawn from each patient, and genomic DNA was extracted from the peripheral leukocytes of the patients using a QIAamp DNA Blood Mini Kit (Qiagen, Hilden, Germany), according to the manufacturer’s protocol. Samples were quantified by spectrophotometry and diluted to 5 ng/μL for polymerase chain reaction (PCR). We performed PCR for these samples and subsequent Sanger sequencing for all exons. The primers were designed to amplify all 17 exons of the *TGFBI* gene. Each PCR was conducted in a 20-μL reaction mixture (Maxime PCR Premix kit; iNtRON Biotechnology, Seongnam, Korea) that contained 100 ng of genomic DNA, 10 pmol of forward and reverse primers, and distilled water. Samples were amplified in 35 cycles of 20 s at 94 °C for denaturing, 15 s at 58 °C for annealing, and 50 s at 72 °C for extension in a 96-well thermal cycler (Applied Biosystems, Lincoln Centre Drive Foster City, CA, USA). To screen out the mutation, we compared the DNA sequence of the proband with a complementary *TGFBI* DNA sequence from GenBank (NC_000005.10). For pediatric patients, genomic DNA was amplified from hair follicles and from buccal cells obtained via cheek-scraping with sterile swabs (Whatman® OmniSwab; GE Healthcare Bio-Sciences, Pittsburgh, PA, USA) according to Buccal Swab Spin Protocol [29,30]. Because a mutation was identified at exon 11 of the proband, we also analyzed exon 11 of *TGFBI* in the remaining family members. Exon 11 was also examined in 100 healthy Korean individuals as a control.

To confirm the maternity and paternity of the proband, the genotypes of amelogenin and 22 autosomal short tandem repeats (A-STRs) were determined for the proband and his parents using the PowerPlex® Fusion System (Promega, Madison, WI, USA). The PCR amplification was performed according to the manufacturer’s instructions with 1 ng of DNA. The PCR-amplified products were separated using capillary electrophoresis on an ABI PRISM 3130xl Genetic Analyzer (Applied Biosystems), and the results were analyzed using GeneMapper ID Software version 3.2 (Applied Biosystems). Shared alleles at each tested loci were identified and the probability of paternity for the trio case was calculated using known allele frequencies for 22 A-STR in Koreans [31].
